# Protein Determination with Molecularly Imprinted Polymer Recognition Combined with Birefringence Liquid Crystal Detection

**DOI:** 10.3390/s20174692

**Published:** 2020-08-20

**Authors:** Maciej Cieplak, Rafał Węgłowski, Zofia Iskierko, Dorota Węgłowska, Piyush S. Sharma, Krzysztof R. Noworyta, Francis D’Souza, Wlodzimierz Kutner

**Affiliations:** 1 Institute of Physical Chemistry Polish Academy of Sciences, Kasprzaka Str. 44/52, 01-224 Warsaw, Poland; mcieplak@ichf.edu.pl (M.C.); ziskierko@ichf.edu.pl (Z.I.); psharma@ichf.edu.pl (P.S.S.); wkutner@ichf.edu.pl (W.K.); 2 Faculty of Advanced Technologies and Chemistry, Military University of Technology, Kaliskiego Str. 2, 00-908 Warsaw, Poland; rafal.weglowski@wat.edu.pl (R.W.); dorota.weglowska@wat.edu.pl (D.W.); 3 Department of Chemistry, University of North Texas, 1155 Union Circle No 305070, Denton, TX 76203-5017, USA; francis.dsouza@unt.edu; 4 Faculty of Mathematics and Natural Sciences, School of Sciences, Cardinal Stefan Wyszynski University in Warsaw, Wóycickiego Str. 1/3, 01-815 Warsaw, Poland

**Keywords:** molecularly imprinted polymer, liquid crystal, human serum albumin

## Abstract

Liquid crystal-based sensors offer the advantage of high sensitivity at a low cost. However, they often lack selectivity altogether or require costly and unstable biomaterials to impart this selectivity. To incur this selectivity, we herein integrated a molecularly imprinted polymer (MIP) film recognition unit with a liquid crystal (LC) in an optical cell transducer. We tested the resulting chemosensor for protein determination. We examined two different LCs, each with a different optical birefringence. That way, we revealed the influence of that parameter on the sensitivity of the (human serum albumin)-templated (MIP-HSA) LC chemosensor. The response of this chemosensor with the (MIP-HSA)-recognizing film was linear from 2.2 to 15.2 µM HSA, with a limit of detection of 2.2 µM. These values are sufficient to use the devised chemosensor for HSA determination in biological samples. Importantly, the imprinting factor (*IF*) of this chemosensor was appreciable, reaching *IF* = 3.7. This *IF* value indicated the predominant binding of the HSA through specific rather than nonspecific interactions with the MIP.

## 1. Introduction

Since Abbott’s group first reported on the use of a 5CB (4-pentyl-4′-cyanobiphenyl) liquid crystal (LC) as the sensing element of a chemosensor for optical determination of the IgG [1,2] and avidin (Av) [1] interaction with biotin, it became apparent that biomolecules deposited on a solid surface may affect the reorientation of the LC molecules [3]. This reorientation generates an optical response seen under a polarized light optical microscope. This phenomenon was used for devising biosensors for the determination of proteins [4,5,6], DNA [7,8,9,10] and heavy metal ions [11]. Moreover, optical birefringence can not only be used for transduction in LC-based protein sensors, but their unique electro-optical and dielectric properties can also be successfully exploited to this end [12]. However, until now, these sensors either lack selectivity, or their selectivity is due to antibody–antigen interactions requiring costly biological reagents, which, moreover, are prone to instabilities.

Human serum albumin (HSA) is an essential component of the protein system of human blood plasma [13]. This protein transports numerous vital compounds in the human body, namely hormones, fatty acids, and drugs [14]. The typical HSA concentration in blood plasma is in the range of 35 to 50 mg/mL (0.53 to 0.75 mM). An abnormal level of HSA in plasma indicates possible diseases, including liver failure, cirrhosis, chronic hepatitis [15], coronary heart disease [16], and multiple myeloma [17]. Its moderate concentration (0.45 to 4.51 µM, i.e., 30 to 300 µg/mL) in urea may indicate microalbuminuria and, at higher (exceeding 4.51 µM) concentrations, albuminuria. Those deficiencies are caused by kidney damage, mainly the result of diabetes and hypertension [18].

Typically, HSA is determined using colorimetry [19]. However, this technique suffers from the overestimation of the HSA concentration and reactions with other proteins. Fluorescence-based methods drawbacks are low selectivity and sensitivity [20]. A stable fluorescence method has been developed, although with quite a restricted linear dynamic concentration range (2–10 µg/mL, i.e., 30–150 nM) [21]. Moreover, an impedimetric immunosensor for HSA has been devised with high sensitivity and a wide linear dynamic concentration range [22]. Unfortunately, this sensor suffered typical problems characteristic of immunosensors, i.e., a lack of stability and a costly preparation procedure.

The imprinting of macromolecular templates (*M*_w_ > 1500 Da) within polymers has been the object of extensive studies [23,24,25,26,27]. Molecularly imprinted polymers (MIPs), with their tailored molecular cavities, enable for the selective recognizing of target analytes via binding in these cavities, similarly to biological receptors or antibodies [28,29]. Therefore, MIPs can be used as selective recognition units in chemosensors. As such, they are often called “plastic antibodies”. Interestingly, the application of LCs in the preparation of MIPs can essentially be beneficial in terms of the lower cross-linker amount that is required [30]. Moreover, the LC-containing MIP films were considered as recognition units in chemosensors for organophosphate [31]. However, no clear advantage over other types of MIPs was indicated.

The combination of the ease of preparation and plausible sensitivity of LC-based sensors with robust and highly selective MIPs may result in a promising method of the fabrication of stable, sensitive, and selective chemosensors. To this end, herein, we have devised and fabricated a new optical LC chemosensor. A film of MIP templated with HSA and deposited by electropolymerization served as its recognition unit. The preliminary results of this study were reported as a poster at the SMCBS’2017 workshop [32]. In the present paper, we show the final results of the studies in this direction. The HSA analyte binding by the MIP affects the orientation of LC molecules. In effect, light intensity passing through the sensing unit is changed, thus allowing for the detection and quantification of the analyte.

The combination of the MIP-based recognition with LC-based transduction offers the possibility of sensor miniaturization with relative ease given the existing LCD technology. Furthermore, it provides the opportunity of signal multiplexing, potentially allowing for parallel analyte determination in multiple samples and the simultaneous determination of various analytes, as well as an “artificial tongue”-type sensor fabrication. It is noteworthy that, for the LC sensor performance, macromolecules (globulins, fibrinogen, etc.) would be the main interferences, and not small molecules, because the former can strongly influence the order of LC molecules in the sensor cell. Thus, globulins and fibrinogen will be the main interferences in the blood plasma samples. However, molecules of all these proteins are much larger than those of the HSA. Therefore, they would not fit the molecular cavities of the MIP. Another target sample, urine, predominantly contains compounds with small molecular mass (urea, creatinine and electrolytes). Possible interferences, e.g., proteins, are typically present in abnormal urine samples. Such samples may contain mainly HSA. Hence, interference here is much less pronounced than for blood plasma samples.

To gain precise control over the HSA imprinting, we used a semi-covalent imprinting strategy introduced in our previous study [33]. The procedure developed herein allowed for determining HSA in the concentration range of 2.2 to 15.2 µM (0.148 to 1 mg/mL) with a limit of detection (LOD) of 2.2 µM (148 µg/mL) and a significant apparent imprinting factor of *IF* = 3.7. Noticeably, the dynamic linear concentration range of the devised chemosensor is broader than that of a commonly used colorimetric sensor. Moreover, the former chemosensor is less susceptible to drawbacks revealed by the latter.

## 2. Materials and Methods

### 2.1. Materials

All chemicals, solvents, and peptides were purchased from Sigma-Aldrich, except 2,2′-bithiophene-5-carboxylic acid (Enamine, Ltd., Monmouty, NJ, USA), as well as *N*-(3-dimethylaminepropyl)-*N*′-ethylcarbodiimide hydrochloride (EDC) and 1-hydroxy-7-azabenzotriazole (HOAt) (TCI, Tokyo, Japan).

Two liquid crystalline materials were used. One was genuine 5-cyanobiphenyl (5CB) with the following parameters: Cr 22.5 N 34.2 Iso; *n*_e_ = 1.7140; *n*_o_ = 1.5297; Δ*n* = 0.1843 at 22 °C and 633 nm [34]. The other material was a liquid crystal eutectic nematic mixture 1658 with the following parameters: Cr < 0 N 116.0 Iso; *n*_e_ = 1.9135; *n*_o_ = 1.5359; Δ*n* = 0.3776 at 20 °C and 633 nm and birefringence Δ*n* = 0.37 at a wavelength of 589.3 nm at 20 °C. Both materials were obtained from the Military University of Technology in Warsaw, Poland.

### 2.2. Instruments

All surface plasmon resonance (SPR) and electrochemical experiments were performed on a dual-channel AUTOLAB ESPRIT SPR spectrometer with a 632 nm laser source (Eco Chemie, Utrecht, The Netherlands) driven by ESPRIT v. 4.4 software of the same manufacturer. The SPR spectrometer worked in the Kretschmann configuration. Its two channels were equipped with three-electrode cells with SPR Au film-coated glass chips serving as the working electrode and two independent sets of Pt wires and Ag wires serving as the counter and quasi-reference electrode, respectively. For electrochemical experiments, both cells were connected to an AUTOLAB PGSTAT20 potentiostat/galvanostat driven by GPES4.5 software.

The MIP films were atomic force microscopy (AFM) (in the Peak Force quantitative nanomechanical mode (QNM)) imaged with a Bruker MultiMode 8 AFM microscope under the control of a Nanoscope V controller and driven by Multimode 8.15 software of the same manufacturer. For imaging, the films were deposited on Au film-coated glass slides. Calibrated ScanAssyst™ Air probes with silicon tips and a force constant of 0.42 N/m and a tip diameter of 10 nm were used.

### 2.3. Procedures

#### 2.3.1. Synthesis of Functional Monomers

Syntheses of the *p*-bis(2,2′-bithien-5-yl)methylalanine functional monomer FM 2 and the 5,5′,5′′-methanetriyltris(2,2′-bithiophene) cross-linking monomer CM are described elsewhere [35,36].

#### 2.3.2. Synthesis of the HSA-Templated Molecularly Imprinted Polymer (MIP-HSA) Film and the Control Non-Imprinted Polymer (NIP) Film

MIP-HSA films were deposited on semitransparent Au film-coated glass slides by oxidative electropolymerization under potentiodynamic conditions. For that, one potential cycle over the potential range of 0 to 1.0 V vs. Ag quasi-reference electrode at a scan rate of 50 mV/s was applied. For the preparation of a solution for the electropolymerization, 1 µL of 3 mg/mL, i.e., 0.045 mM, solution of the functional monomer-derivatized HSA was added to 100 µL of the acetonitrile solution of 1 µM 5,5′,5′′-methanetriyltris(2,2′-bithiophene) cross-linking monomer CM and 10 mM tetrabutylammonium perchlorate supporting electrolyte. The procedure of HSA derivatization with two different functional monomers, namely 2,2′-bithiophene-5-carboxylic acid (FM 1) and *p*-bis(2,2′-bithien-5-yl)methylalanine (FM 2) was described previously [33].

The control NIP films were prepared in the same way as the MIP-HSA films but in the absence of HSA. The appropriate amounts of free FM 1 and FM 2 were added to the solution for the electropolymerization instead of the (functional monomer-derivatized) HSA.

After MIP preparation, the HSA template was removed from the MIP-HSA film by immersing the Au film-coated slides in 30% NaOH for 45 min.

#### 2.3.3. SPR Determination of HSA

All SPR measurements were performed at room temperature, 20 (±1) °C, in a PBS (pH = 7.4) solution using an electrochemical SPR setup based on a dual-channel AUTOLAB ESPRIT SPR spectrometer (Eco Chemie, Utrecht, The Netherlands) described in Section 2.2 above. After the HSA solution sample injection, a cyclic voltammetry experiment (30–50 cycles, from 0 to 0.50 V vs. Ag quasi-reference electrode, at a scan rate of 50 mV/s) was performed until the SPR signal became stable.

#### 2.3.4. Procedure of the HSA Determination Using Liquid Crystal Optical Cells

LC optical cells were prepared, according to the procedure described below, to determine HSA. First, the MIP film-coated Au slides were immersed in an HSA solution of PBS (pH = 7.4) for 12 h at 4 °C to assure analyte binding. Simultaneously, (homeotropic alignment)-coated glass slides were prepared, as follows. A DMF solution (1 wt.%) of SE 1211 (Nissan Chemicals, Tokyo, Japan.) was spin-coated onto glass substrates with an indium-tin-oxide (ITO) conductive transparent layer. Then, the solvent was evaporated at 130 °C for 1.5 h. The ellipsometrically estimated thickness of the deposited homeotropic alignment layer was 20 to 25 nm.

**Scheme 1:**
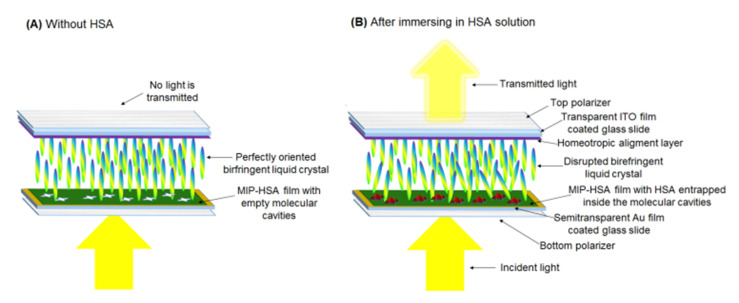
Illustration of the liquid crystal optical cell with the human serum albumin molecularly imprinted polymer (MIP-HSA) recognition film (**A**) without HSA and (**B**) after immersing in an HSA solution.

Subsequently, the homeotropic LC optical cells were constructed (Scheme 1). For that, two substrates were used. The ITO layer, featuring a homeotropic alignment layer, was one of them. The other was a semitransparent Au film-coated glass slide with the deposited MIP-HSA or NIP film. A 5-µm thick glass spacer controlled the distance between these substrates. The empty cells were filled with selected LC by capillary suction. Two LC materials were used. One was 5-cyanobiphenyl (5CB), and the other was LC eutectic nematic mixture 1658 with higher birefringence. Parameters of both LCs are given in the Supporting Information.

The LC optical cells were placed between crossed polarizers in an Olympus BX51 polarizing microscope (Olympus) operating in transmission mode. Then, the white light of a halogen lamp was transmitted through this setup. A square-wave electric field of an amplitude of *E* = 2 MV/m and 1 kHz frequency was applied to the cell. This field was generated by a Hewlett-Packard HP 33120A function generator and a voltage amplifier, and monitored by an HP 346013 oscilloscope of the same manufacturer. All the measurements were performed at room temperature.

The freeware Image-Pro Plus, digital image analysis software, was applied to quantify the optical textures. For that purpose, each registered image intensity was quantified by summing up the total RGB values of all pixels within a square, and then this sum was divided by the region area. The obtained value is here referred to as light intensity.

## 3. Results and Discussion

### 3.1. MIP and NIP Film Deposition and Characterization

HSA derivatized with the functional monomers was used for the deposition of MIP-HSA films on the semitransparent Au film-coated glass slides. For that, our semi-covalent HSA imprinting procedure was modified [33]. In brief, HSA molecules were chemically modified with monomers FM1 and FM2. Then, the modified HSA was electropolymerized in the presence of the cross-linking monomer. Finally, the HSA was extracted from the resulting MIP with NaOH. In the case of the present research, deposition conditions were optimized to obtain thin (10–20 nm) MIP-HSA films (Figures S1, S2 and Table S1).

**Figure 1:**
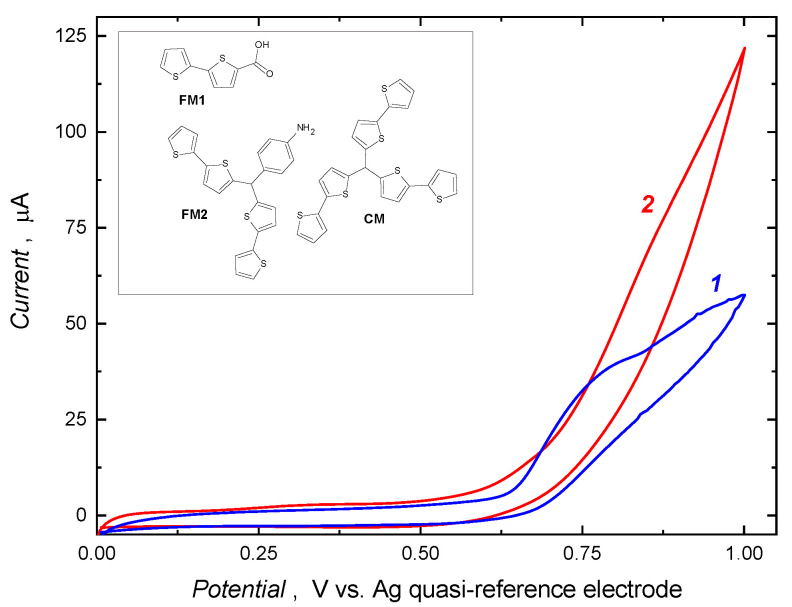
Current–potential curves of one-cycle potentiodynamic electropolymerization leading to deposition of the film of (1) MIP and (2) non-imprinted polymer (NIP) on the surface plasmon resonance (SPR) chip from a mixture of acetonitrile solutions of 1 µL of 3 mg/mL, i.e., 0.045 mM, (functional monomer)-derivatized HSA, as well as 100 µL of 1 µM 5,5′,5′′-methanetriyltris(2,2′-bithiophene) crosslinking monomer (CM) and 10 mM tetrabutylammonium perchlorate. Inset shows structural formulas of functional monomers FM 1 and FM 2, as well as the cross-linking monomer CM.

Figure 1 presents an example of the current–potential curves recorded during the MIP and NIP films deposition by potentiodynamic electropolymerization. Thiophene moieties are electro-oxidized herein at ~0.70 V vs. Ag quasi-reference during both MIP and NIP film deposition. However, the anodic current for MIP film deposition was lower (curve 1 in Figure 1) than that for the NIP film (curve 2 in Figure 1). This effect might arise from the slow diffusion of bulky derivatized HSA molecules on the one hand, and the partial blocking of the electrode surface by large non-conducting protein molecules on the other.

Moreover, the deposition of the polymer film was confirmed by changes in the SPR signal recorded during MIP and NIP film deposition (Figure S3, Supporting Information). Interestingly, the addition of a solution (functional monomer)-derivatized HSA to the SPR cell leads to a pronounced, exceeding 2 degrees, increase in the SPR angle. This increase implies strong adsorption of the derivatized HSA on the SPR chip surface.

**Figure 2:**
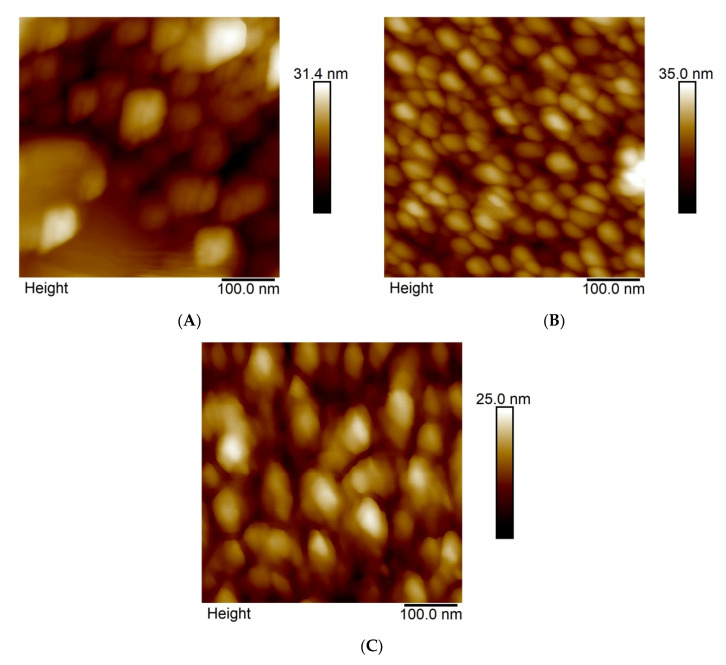
Atomic force microscopy (AFM) images (500 × 500 nm^2^) of the (**A**) MIP, (**B**) extracted MIP, and (**C**) NIP film deposited on Au film-coated glass slides by electropolymerization under potentiodynamic conditions.

The MIP-HSA film was AFM imaged to unravel its morphological differences before and after HSA extraction (Figure 2). Apparently, the MIP-HSA surface is slightly non-uniform. The gold substrate grains are enveloped in the polymer film (Figure 2A). After HSA extraction, the film becomes more uniform (Figure 2B). The AFM-determined thickness of the MIP-HSA film is 33.4 (±4.7) and 16.1 (±1.6) nm before and after HSA extraction, respectively. The morphology of the NIP film slightly differs from that of the MIP film (Figure 2C). The deposited polymer shells are more connected in the case of the NIP film, thus leading to more deformed grains. This effect is in accord with higher currents (Figure 1), i.e., a larger amount of NIP film deposited during electrochemical polymerization. After HSA extraction, the NIP film thickness is slightly lower than that of the MIP film, equaling 11.7 (±1.4) nm. Nanomechanical measurements (Figure S4 and Table S2) indicate that the films are rather uniform and rigid, with negligible deformation. Interestingly, the Si tip adheres substantially stronger to the non-extracted MIP film than to the HSA-extracted MIP or NIP film. This adhesion difference may indicate the formation of a weakly bound film on the surface of the MIP, which is then removed during the extraction.

### 3.2. SPR Sensor Preparation and Testing

SPR was used for the preliminary testing of HSA binding by the MIP-HSA film. Details of the SPR determination procedure are presented in Section 2.3.3 above. The small thickness (~16 nm) of the deposited MIP film was a reason why the imprinted cavities were located very close to the signal-transducing metal surface. SPR is sensitive to binding phenomena that occur in the close vicinity (≤ 50 nm) of the metal surface. Therefore, it was possible to observe the SPR signal change corresponding to the HSA binding in the MIP film. Interestingly, the SPR signal decreased with the increase in the HSA concentration in solution. Typically, protein binding leads to an SPR signal increase because of an increase in the refraction index in the proximity of the metal surface. However, here we had to take into consideration changes in the MIP-HSA film structure/morphology during HSA binding. This effect may originate from different phenomena. Namely, HSA analyte binding in the MIP may induce changes in the polymer electrical properties.

Moreover, the polymer itself may shrink or expand during HSA binding. Furthermore, HSA molecules can replace solvent molecules in the imprinted cavities. All these effects may result in a net negative shift of the resonance angle of incident light, i.e., in decreasing the SPR signal.

**Figure 3:**
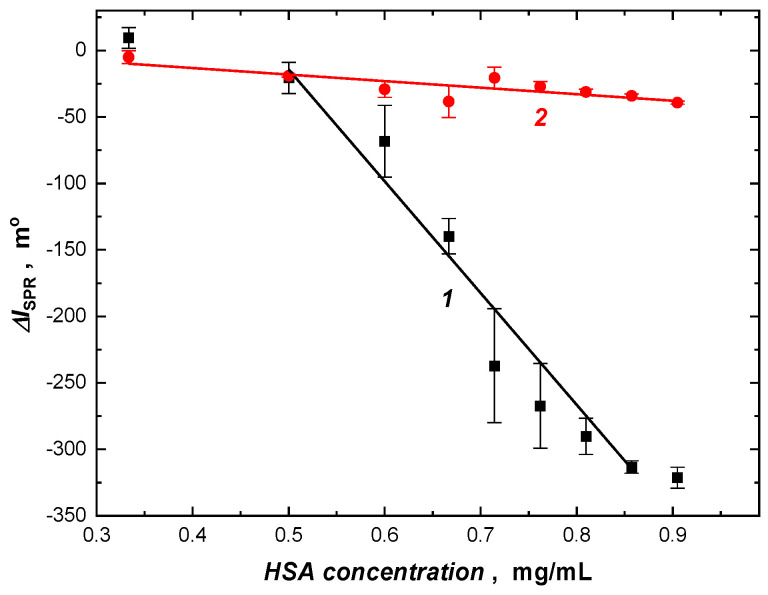
The SPR calibration plots for human serum albumin (HSA), for the (1) MIP and (2) NIP film-coated SPR chips in 0.1 M PBS (pH = 7.4).

In the SPR determinations, the HSA-extracted MIP-HSA film-coated chip linearly responded to the HSA concentration in solution in a rather narrow concentration range of 0.5 to 0.85 mg/mL, i.e., 7.52 to 12.75 µM (Figure 3), obeying the linear regression equation of Δ*I*_SPR_, m° = 4.05 × 10^2^ (±28.8) − 8.4 × 10^2^ (±36.0) *c*_HSA_, mg/mL with a correlation coefficient of *R*^2^ = 0.9909. Because of the calibration curve slope change observed at ~0.5 mg/mL (~7.52 µM), the limit of detection, calculated as 3σ/(calibration curve slope), was estimated to be ~6.8 µM (or ~450 µg/mL) of the HSA. The apparent imprinting factor, which was calculated as the ratio of slopes of the calibration curves for MIP and NIP, was very high, *IF*_SPR_ = 17. At the HSA concentrations exceeding 0.85 mg/mL (12.75 µM), the SPR signal progressively deviated from linearity, reaching its saturation at ~0.9 mg/mL (13.50 µM). This effect was not observed for the NIP film-coated SPR chip, where the decreasing trend continued. This effect indicates that the MIP recognition film became saturated with HSA at concentrations exceeding ~0.85 mg/mL.

### 3.3. LC Sensor Preparation and Testing

When the voltage is applied between the transparent ITO electrode and the semitransparent Au film-coated glass slide, the electric field is developed across the LC optical cell. Then, the director of the dielectrically positive (with the electric permittivity anisotropy, Δ*ε* > 0) liquid crystalline medium aligns parallel to the electric field applied (Scheme 1). If then the cell is illuminated with light polarized by the bottom polarizer, the liquid crystal layer does not change its polarization plane. It is then blocked by the top crossed polarizer and the optical cell appears dark. When the protein molecules are bound to the MIP film, the alignment of LC molecules is disturbed, thus making the local optical axis no longer parallel to the light path. Then, the polarized light is partially transmitted through the top polarizer and recorded as an optical signal. The fraction of light passing through this second polarizer is directly related to phase retardation, as follows [11]:

*δ* = 2π*d*∆*n*/*λ* (1)

where *d* is the thickness of the disturbed LC layer, *λ* is the wavelength of the incident light, and Δ*n* is optical birefringence.

When the LC optical cell is placed between crossed polarizers, the intensity of the light, *I*, emerging from the top polarizer is:

*I* ∝ *I*_0_cos^2^*δ* (2)

according to Malus’s law. *I*_0_ is the intensity of incident light. Apparently, the intensity of the transmitted light can be enhanced if an LC with higher optical birefringence is used (Scheme 1). Herein, the abovementioned technique was used to determine the amount of HSA captured by the HSA-extracted MIP-HSA film as the light intensity passed through the LC cell is proportional to number of the HSA molecules bound by the MIP film. The larger the number of bound HSA molecules, the stronger the disturbance of the LC molecule organization.

For testing the LC determination of HSA, we used the optical cell described above. In fact, we tested two LCs with different birefringences to reveal the influence of the birefringence parameter on the sensitivity of the MIP-HSA LC chemosensor. An LC with higher birefringence, such as the 1658 LC, may enhance the chemosensor sensitivity. This is because phase retardation changes in the presence of HSA are more pronounced compared to those of an LC with lower birefringence, such as the 5CB LC.

**Figure 4:**
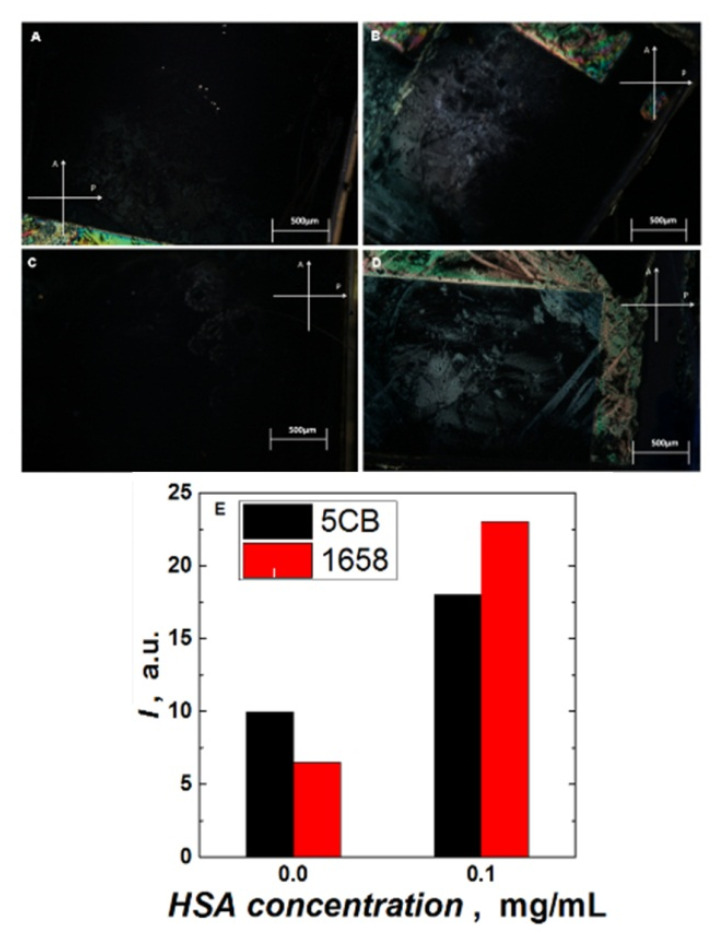
Optical textures of the (**A**,**B**) 5CB and (**C**,**D**) 1658 liquid crystals (LCs) recorded for MIP-HSA film-coated slides after immersion (**A**,**C**) PBS (pH = 7.4) and (**B**,**D**) 1.5 µM, i.e., 0.1 mg/mL HSA in PBS (pH = 7.4). (**E**) Histogram comparing intensities of light passing through the 5CB and 1658 LCs that were previously immersed in PBS (pH = 7.4) with and without HSA.

We examined this hypothesis by binding HSA in imprinted cavities of the MIP-HSA thin film deposited on the semitransparent Au film, followed by its detection with either the 1658 or 5CB LC. In both cases, an electric field of 2 MV/m was applied. Images of optical textures and the corresponding total intensities recorded for the 5CB or 1658 LC in contact with the extracted MIP-HAS films and the MIP-HSA films after equilibration with solution of the HSA are shown in Figure 4. For both LCs, there was an increase in the intensity of light passing through the LC optical cell if the MIP-HSA film was immersed in the HSA solution. Noticeably, the 1658 LC gives a more pronounced increase in the passing light intensity than the 5CB does for a given HSA concentration. Thus, as expected, this indicates that a detectability can be higher whenusing a liquid crystal with higher birefringence. Therefore, subsequent experiments were performed using the 1658 LC.

**Figure 5:**
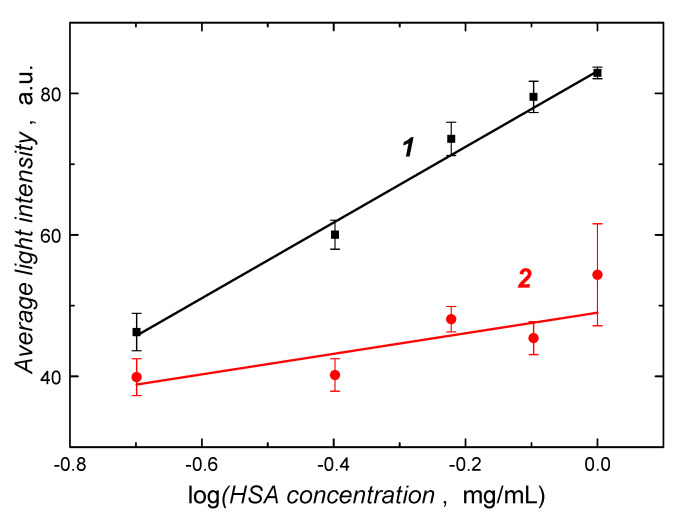
The calibration plots for human serum albumin (HSA) analyte in 0.1 M PBS (pH = 7.4) constructed using 1658 (liquid crystal eutectic)-containing optical cells with (1) MIP-HSA and (2) NIP film-coated semitransparent Au film-coated glass slides.

The HSA calibration curves constructed with the 1658 eutectic LC optical cell, either with the HSA-extracted MIP-HSA or NIP film-coated semitransparent Au film-coated glass slides, are shown in Figure 5. Apparently, the LC MIP-HSA chemosensor linearly responded to the logarithm of HSA concentration in solution in the range of 0.14 to 1 mg/mL, i.e., 2.2 to 15.15 µM, obeying the linear regression equation of Δ*I* = 83.14 (±0.68) + 53.49 (±2.97) × log *c*_HSA_, mg/mL with a correlation coefficient *R*^2^ = 0.9954. At a signal-to-noise ratio of 3, the LOD was 148 µg/mL (2.2 µM) HSA.

It is interesting here to compare the results obtained by using SPR transduction (Figure 3) with those obtained with the LC transduction (Figure 5). First of all, a well-established SPR technique corroborates that the devised MIP film is indeed capable of binding the HSA and that the selective imprinted cavities are formed successfully. The latter conclusion follows from a high apparent imprinting factor determined using SPR. The SPR technique is primarily sensitive to changes in the refractive index of a film adjacent to the Au film-coated sensor chip, close to its surface, and, therefore, is mainly dependent on the molecular mass of the adsorbing species. It is much less sensitive to other factors. Thus, the SPR studies allowed us to test the devised MIP film before combining it with the new and untested transduction technique. Importantly, both transduction techniques used showed responses in somewhat similar concentration ranges. However, the LC sensor was more sensitive in a lower concentration range than the SPR sensor, as confirmed by its lower LOD. It shows that even a small alteration to the LC molecule order results in a detectable change of the light intensity. It is noteworthy that, the apparent imprinting factor determined with the LC transduction technique was lower than that with the SPR technique, equaling *IF*_LC_ = 3.7. Evidently, the LC transduction technique is more sensitive to nonspecific HSA adsorption on the polymer film surface than the SPR technique. Presumably, this adsorption would also affect the orientation of LC molecules, thus leading to an increase in the intensity of light passing through the LC optical cell.

## 4. Conclusions

We devised, fabricated, and tested a new optical chemosensor for the selective detection and determination of HSA. For that, we molecularly imprinted HSA in a thin (~16-nm thick) conducting polymer film. This film was deposited by potentiodynamic electropolymerization on a semitransparent Au SPR chip. First, the HSA binding was confirmed by SPR to check the MIP film performance. The SPR studies confirmed that the MIP film contained the HSA binding cavities. Subsequently, the birefringence of an LC was used as a transduction technique for the optical determination of the HSA analyte. To our best knowledge, this is a unique successful combination of MIP recognition with birefringence LC detection. Furthermore, we have confirmed that the LC molecules with higher birefringence are better suited for LC sensor construction because of the higher contrast of the cell before and after the HSA binding. This chemosensor is well suited to devising sensor matrices for parallel and simultaneous determinations of various proteins. Moreover, it can readily be miniaturized. The LC MIP-HSA chemosensor response to the HSA concentration in solution was linear in the range of 2.2 to 15.2 µM with the LOD of 2.2 µM HSA. This detection limit allowed us to use this chemosensor for HSA determination in biological samples, including urine and blood plasma. The HSA concentration expected in blood plasma samples can be determined by using the LC sensor after dilution. Furthermore, our chemosensor response range covered the concentrations of HSA in urine that are considered as borderline between normal levels and those regarded as symptomatic of microalbuminuria and, especially, albuminuria. The sensitivity of the MIP and NIP chemosensors to HSA were compared, and the apparent imprinting factor, *IF*_LC_ = 3.7, was determined. It is noteworthy that, depending on the transduction technique used, the imprinting factor may be influenced by other physical effects accompanying the analyte binding (e.g., polymer chain rearrangements inducing additional refractive index change, etc.), thus leading to different apparent imprinting factors for different transduction techniques.

## 5. Patents

Polish patent application P.418746 of 19 September 2016.

## Supplementary Materials

The following are available online at https://www.mdpi.com/1424-8220/20/17/4692/s1, Figure S1: Cyclic voltammograms recorded during MIP film electrodeposition; Figure S2: SPR reflectivity curves recorded for different MIP films; Table S1: Summary of the film deposition condition optimization; Figure S3: Changes of the SPR signal with time recorded during deposition of the polymers; Figure S4: Adhesion and deformation maps of MIP and NIP films; Table S2: Selected properties of the electrodeposited films derived from AFM and nanomechanical images.
