# Supplementary material for: Protein Determination with Molecularly Imprinted Polymer Recognition Combined with Birefringence Liquid Crystal Detection
Source: Sensors (Basel). 2020 Aug 20;20(17):4692. doi: 10.3390/s20174692 (PMC7547379; doi:10.3390/s20174692)
Supplement: Supplementary file 1 [file sensors-20-04692-s001.docx]

Supporting Information

Protein Determination with Molecularly Imprinted Polymer Recognition Combined with Birefringence Liquid Crystal Detection

Maciej Cieplak ^1,†^, Rafał Węgłowski ^2,†^, Zofia Iskierko ^1^, Dorota Węgłowska ^2^,
Piyush S. Sharma ^1^, Krzysztof R. Noworyta ^1,^*, Francis D’Souza ^3^ and
Wlodzimierz Kutner ^1,4^

^1^ Institute of Physical Chemistry Polish Academy of Sciences, Kasprzaka Str. 44/52, 01-224 Warsaw, Poland;

[mcieplak@ichf.edu.pl](mailto:mcieplak@ichf.edu.pl) (M.C.); [ziskierko@ichf.edu.pl](mailto:ziskierko@ichf.edu.pl) (Z.I.); [psharma@ichf.edu.pl](mailto:psharma@ichf.edu.pl) (P.S.S.) [wkutner@ichf.edu.pl](mailto:wkutner@ichf.edu.pl) (W.K.)

^2^ Faculty of Advanced Technologies and Chemistry, Military University of Technology, Kaliskiego Str. 2, 00-908 Warsaw, Poland; [rafal.weglowski@wat.edu.pl](mailto:rafal.weglowski@wat.edu.pl) (R.W.); [dorota.weglowska@wat.edu.pl](mailto:dorota.weglowska@wat.edu.pl) (D.W.)

^3^ Department of Chemistry, University of North Texas, 1155 Union Circle No 305070, Denton, TX 76203-5017, USA; [francis.dsouza@unt.edu](mailto:francis.dsouza@unt.edu)

^4^ Faculty of Mathematics and Natural Sciences, School of Sciences, Cardinal Stefan Wyszynski University in Warsaw, Wóycickiego Str. 1/3, 01-815 Warsaw, Poland

* Correspondence: knoworyta@ichf.edu.pl; Tel.: +48-22-343-3217

† Both authors contributed equally to this work.

Received: 18 July 2020; Accepted: 17 August 2020; Published: date

Table of Contents

1. Potentiodynamic curves for MIP film deposition by electropolymerization S-2

2. SPR reflectivity curves for different MIP films S-3

3. Summary of optimization the film deposition conditions S-4

4. Surface plasmon resonance signal recorded during MIP and NIP film deposition S-5

5. Adhesion and deformation mapping of the MIP and NIP films S-6

6. MIP and NIP film properties determined by AFM and nanomechanical imaging S-7

1. Potentiodynamic Curves for MIP Film Deposition by Electropolymerization

**Figure S1.** Potentiodynamic curves for MIP film deposition by electropolymerization during (**a**) one, (**b**) two, or (**c**) three potential cycles. The potential ranged from 0 to 1.00 V vs. Ag quasi-reference electrode. The potential scan rate was 50 mV/s. The polymerization solution was 1 µM in cross-linking monomer **3**, 30 µg/mL in human serum albumin-functional monomers **1** and **2** conjugate, and 10 mM in tetrabutylammonium perchlorate in the acetonitryle and PBS (pH = 7.4) 100 : 100 (*v* : *v*.) mixture. The inset to (**a**) presents structural formulas of the monomers.

2. SPR Reflectivity Curves for Different MIP Films

**Figure S2.** SPR reflectivity curves for (1, 2) bare gold-coated SPR chips as well as Au film-coated SPR chips coated with the MIP films of (3) 33.4, (4) 53.2, and (5) 94.8 nm thickness. Measurements were performed in (1) air and using (2-5) PBS (pH = 7.4).

3. Summary of Optimization the Film Deposition Conditions

**Table S1.** Parameters used for the optimization of the MIP film deposition conditions and the summary of the results.

| **Procedure number** | **Cross-linking Monomer Concentration, µM** | **Supporting Electrolyte Concentra-tion,**  **mM** | **Applied Potential Range,**  **V vs. Ag** | **Number of Cycles** | **Electric Charge Passed,**  **µC** | **Film Thick-ness,**  **nm** | **SPR Signal** | **Color of the Deposited Film** |
| --- | --- | --- | --- | --- | --- | --- | --- | --- |
| 1 | 1 | 10 | 0–1.00 | 1 | 159.2 | 33.4 (±4.7)^a^  16.1 (±1.6)^b^ | Clear SPR signal | Transpa-rent |
| 2 | 1 | 10 | 0–1.00 | 2 | 253.5 | 53.2^c^ | Broad signal. No clear mini-mum of the reflectance | Uniformly pink |
| 3 | 1 | 10 | 0–1.00 | 3 | 451.9 | 94.8^c^ | No SPR resonance | Non-uniform, from pink to dark-blue |
| 4 | 10 | 100 | 0–1.20 | 50 | - | 250–500^b^ | - | - |
| 5 | 0.5 | 10 | 0–1.00 | 1 | - | - | - | - |

^a^ Film thickness determined with AFM.

^b^ Film thickness after HSA extraction determined using AFM.

^c^ Film thickness estimated based on the charge passed.

4. Surface Plasmon Resonance Signal Recorded during MIP and NIP Film Deposition

**Figure S3.** Changes in the SPR signal with the time recorded during deposition of the film of (*1*) MIP and (*2*) NIP by electropolymerization under potentiodynamic conditions. Compositions of the solutions are indicated at curves.

^a^ (TBA)ClO_4_ – tetrabutylammonium perchlorate.

5. Adhesion and Deformation Mapping of the MIP and NIP Films


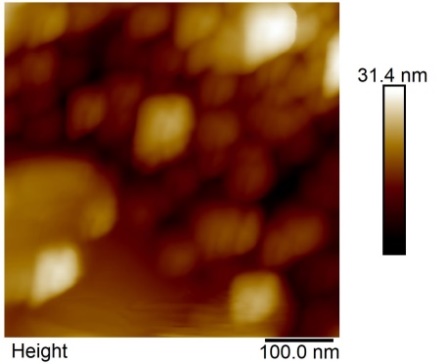

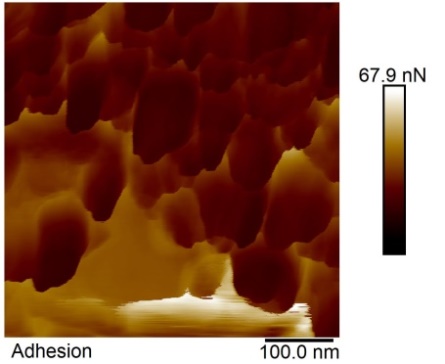

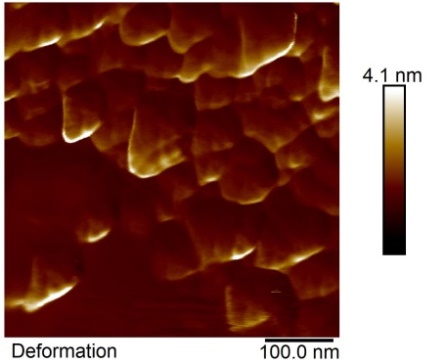


**a’’**

**a’**

**a**


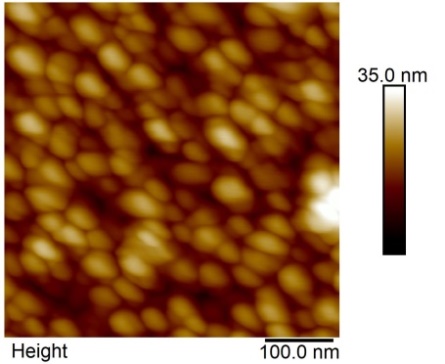

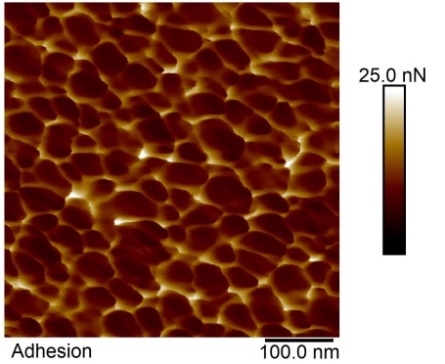

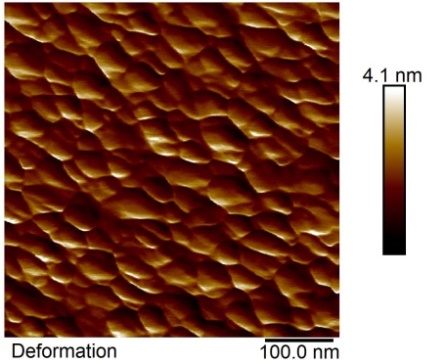


**b’’’**

**b’**

**b**


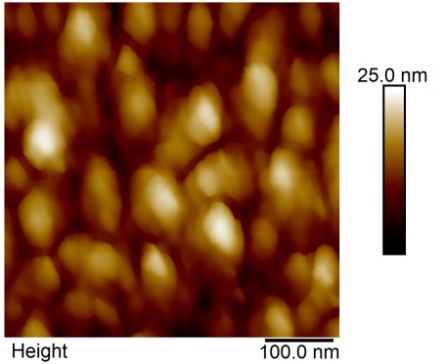

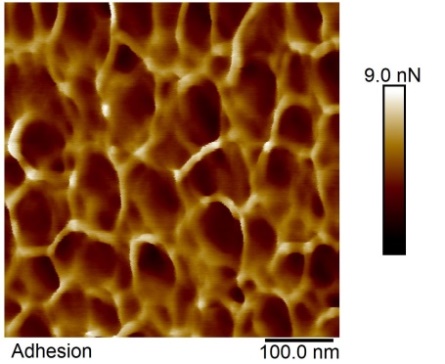

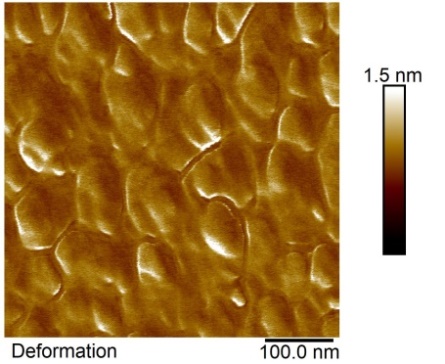


**c’’**

**c’**

**c**

**Figure S4.** Adhesion and deformation maps (500 × 500 nm^2^) of the (a, a’, and a’’) MIP film, (b, b’, and b’’) HSA extracted MIP film, and (c, c’, and c’’) NIP films deposited by electropolymerization on Au film-coated glass slides.

6. MIP and NIP Film Properties Determined by AFM and Nanomechanical Imaging

**Table S2.** Selected properties of MIP and NIP films derived from the AFM and nanomechanical images.

| **Film** | **Thickness, nm** | **Roughness, *R*_a_, nm** | **Adhesion, nN** | **Deformation, nm** |
| --- | --- | --- | --- | --- |
| MIP before HSA extraction | 33.4 ± 4.7 | 3.7 ± 1.2 | 16.5 ± 6.0 | 0.0 ± 0.4 |
| MIP after HSA extraction | 16.1 ± 1.6 | 4.3 ± 1.1 | 5.0 ± 2.4 | 0.0 ± 0.5 |
| NIP | 11.7 ± 1.4 | 2.7 ± 0.2 | 4.2 ± 1.1 | 0.0 ± 0.4 |
